# Patterns in the transmission of traditional ecological knowledge: a case study from Arnhem Land, Australia

**DOI:** 10.1186/s13002-020-00403-2

**Published:** 2020-09-14

**Authors:** Aung Si

**Affiliations:** 1 Institute for Linguistics, University of Cologne, 50923 Cologne, Germany; 2 The MARCS Institute for Brain, Behaviour and Development, Western Sydney University, Locked Bag 1797, Penrith, NSW 2751 Australia

**Keywords:** Kune, Language endangerment, Knowledge transmission, Indigenous, Aboriginal, Hunter-gatherer, Lifestyle change

## Abstract

**Background:**

The loss of traditional ecological knowledge in endangered language communities is a cause of concern worldwide. Given the state of current knowledge, it is difficult to say whether language and TEK transmission levels are correlated, i.e. whether the erosion of one is accompanied by erosion of the other. This case study, focusing on a small Indigenous language from northern Australia, represents a first step towards a systematic investigation of this question.

**Methods:**

Speakers of the language Kune (which is currently being transmitted to small children in the community) were asked to identify and name a number of common birds and plants known to occur on Kune traditional lands, through a series of audiovisual stimuli.

**Results:**

There was a weak correlation between speaker age and performance for the plant naming task, but not for the birds. Analysis of the ethnotaxa that were or were not named by individual participants showed that only a small number of plants and birds (approx. 13% and 7% respectively) were known to all participants, while many (approx. 30% and 26% respectively) could only be named by one participant, i.e. the oldest. Edible ethnotaxa were common among the plants and birds that could be named by many people. There was a tendency among younger speakers to use a single umbrella term to label similar-looking species from large genera, such as *Acacia*, whereas older people would have had distinct labels for each species.

**Conclusions:**

Performance in the plant and bird naming tasks was lower than expected for a community where language transmission to younger generations is high. The loss of certain plant and bird names from the active lexicons of some younger Kune speakers may be due to lifestyle change, particularly in terms of food habits, or due to inter-individual differences in life histories. Differences between the transmission of plant and bird names may be due to more frequent interactions with edible plants, as compared to birds.

## Introduction

The endangerment of small, non-literary languages around the world, along with their unique knowledge systems, is a source of great concern [1–4]. The loss of a community’s traditional ecological knowledge (TEK; a community’s knowledge of local plants, animals and ecological relationships) is particularly worrying, not just for the communities concerned, but also for documentary linguists and conservation biologists who strive for the preservation of endangered languages and biological species respectively. Much has been written in the ethnobiological literature about the interrelatedness of biological and cultural (including linguistic) diversity; one of the key findings of this endeavour is that geographical areas of high biological diversity and high cultural diversity happen to coincide globally (reviewed in [5]). It has also been noted that the endangerment of one form of diversity is usually accompanied by endangerment of the other, making it possible to enumerate common threats to both biological and cultural richness [6, 7].

Since the language spoken by a community is inextricably linked with its culture, and since language forms a unique repository of a community’s traditional knowledge [1, 8], it is not surprising that calls for safeguarding the continued transmission of small, endangered languages have gained momentum in recent decades, with the aim of halting the decline in biocultural diversity worldwide [9]. The importance of language in maintaining TEK is recognised by linguists and ethnobiologists alike, and there is a general consensus that both need to be kept at healthy levels for biocultural diversity to flourish. However, the precise nature of the links between these phenomena are often not clearly articulated, but only indirectly implied. This is probably due to a lack of sufficient empirical data from local-scale studies from around the world, an urgent need that has been recognised for some time [5]. For instance, Pretty et al. [6], list ‘language erosion and loss’ as a threat to both biological and cultural diversity (p. 105), but also state elsewhere in the same paper that ‘diverse languages and knowledge bases are threatened today by the dual erosion of biological and cultural diversity’ (p. 104). Both statements are by themselves unproblematic, but taken together, imply conflicting attitudes towards causality. This strongly suggests that the exact linkages and causal mechanisms (if any) between the endangerment of language, knowledge and biocultural diversity are still unclear, and need to be elucidated by further research. Indeed, real-world empirical studies indicate that these phenomena can interact in complex ways, and that it is as yet difficult to make robust cross-cultural and cross-regional generalisations about correlations among the phenomena. Following are some illustrative examples. Cristancho and Vining [10] investigated perceptions of TEK loss in younger generations in two villages in Colombia and Guatemala, and found a high level of attrition in both, partly attributed to language shift (but they note that there were similar levels of environmental richness at both sites, and differing levels of language shift). McCarter and Gavin [11] also reported a perceived loss of TEK in three villages in Vanuatu, but neither language shift nor ecological change were blamed by the respondents. In contrast, environmental degradation brought about by deforestation and mining, and exacerbated by an influx of weeds and feral animals, was held partly responsible for TEK and cultural erosion in several language communities of Cape York in northern Australia [12].

Many factors have been shown to cause a loss of language and TEK, with significant overlap between the two. Grenoble [13] identifies four broad categories of factors responsible for language loss: urbanisation (e.g. increased exposure to broadcast media and the internet), globalisation (e.g. pressure to learn an international lingua franca), social dislocation (e.g. social disadvantage faced by minority groups) and cultural dislocation (e.g. loss of indigenous culture through domination by a more powerful group). A language shift scenario such as mass emigration may be the result of such factors, although Himmelmann [14] warns that no single factor can guarantee language shift, and that language shift, when it occurs, is likely attributable to a constellation of factors. The loss of TEK, similarly, has been shown to be caused by factors such as mainstream schooling, religious conversion, changes in land use, introduction of a market economy, and industrialisation and globalisation processes, among others (reviewed in [15]). Given the apparent similarities between the two lists presented above, it is tempting to conclude that the presence of one or more shared factors in a community (mainstream schooling and urbanisation, for instance) should have a simultaneous, negative impact on both language and TEK. However, there are conflicting reports on the association between language endangerment and TEK transmission in various language communities. On the one hand, it is possible for TEK to remain strong, even in the face of severe language loss, as in the case of Ixatec (Mexico) [16]. On the other hand, loss of an ancestral language (e.g. Marra, northern Australia) and shift to a contact language (in this case, Kriol) can lead to a decline in TEK among the younger generation, even when knowledge of other domains, such as kinship, remains relatively intact [17]. Even more surprisingly, there are documented cases of TEK erosion among people who speak their mother tongue fluently, such as the Tohono O’odham of the southwestern United States [18].

This paper presents a local-scale case study of TEK in an extended family group, who are speakers of the Australian Indigenous language Kune. Language transmission in this community is strong, and even little children speak their mother tongue fluently (almost exclusively, when with their caregivers) in addition to English. Does it follow that the transmission of TEK in this community should also be at a high level, in parallel with language transmission? If so, that would suggest that the factors safeguarding language maintenance and transmission are also working in favour of knowledge maintenance and transmission. If, however, TEK transmission shows signs of weakening, it would indicate that different pressures are acting on TEK and language in this community, and that different strategies may be required for safeguarding them. This study represents the first in a planned series of investigations in four countries of the Asia-Pacific, with the aim of systematically exploring the relationship between language endangerment and TEK endangerment in multiple language communities. The same data collection methods and analytical tools will be used in all target communities (with far bigger sample sizes than in the present study), which should help to minimise some of the problems, relating to comparability, discussed in [19]. It is hoped that this research will inform decisions on the part of community organisations, academics and policy makers who wish to safeguard, document or revitalise language and TEK, by enabling targeted interventions for either, or both, as required.

## Methods

### Field site

**Fig. 1:**
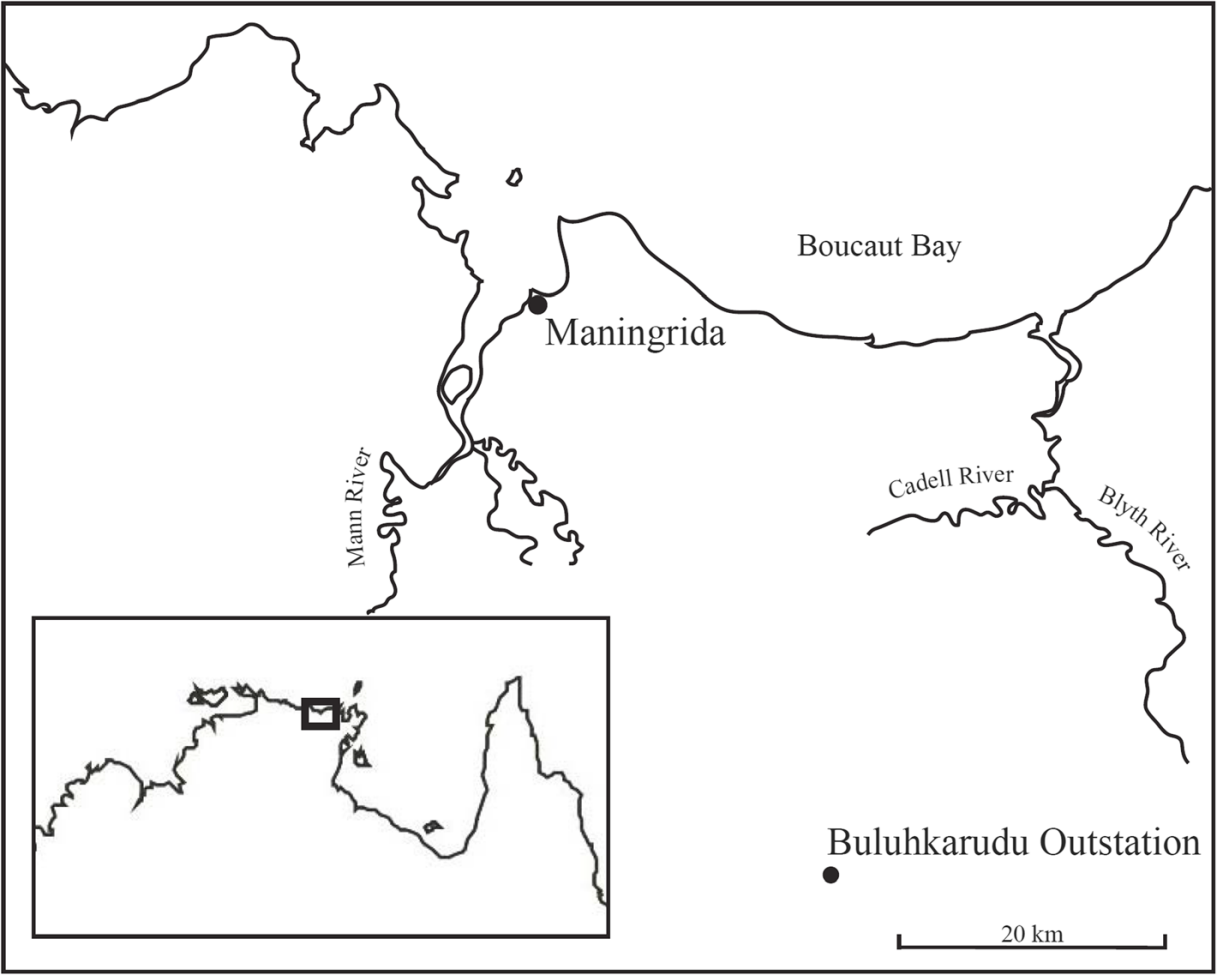
The location of Maningrida and Buluhkaduru in northern Australia

This study was carried out as part of a language documentation project focusing on the endangered Kune language (Non-Pama-Nyungan group, Gunwinyguan family), spoken in north-central Arnhem Land in the Northern Territory, Australia. The total speaker population of Kune is estimated to be around 180 [20]. Although the author has been working with Kune speakers since late 2013, the data for the present study were collected during a 3-week field trip in September 2019. Data collection occurred at Buluhkaduru Outstation (Fig. 1), a traditional homeland of the Kune people, which lies approximately 50 km SSE of the remote coastal town of Maningrida. Kune speakers are inland, or ‘freshwater’ people, which naturally determines the plant and animal species that they are familiar with. Arnhem Land and the neighbouring world-heritage-listed Kakadu National Park are well managed by Indigenous ranger organisations, but in recent years, threats such as changed bushfire regimes, mining and invasive species have led to noticeable losses of local biodiversity. Prominent examples include a reduction in healthy stands of the fire-sensitive native cypress *Callitris intratropica* [21] and a drastic reduction in numbers, or in some cases local extinction, of small, iconic mammals such as the Northern Quoll and the Northern Brown Bandicoot [22].

Arnhem Land is a highly multilingual region, and speakers from many language groups reside in Maningrida and its surrounding outstations (small settlements on traditional lands). Typically, people prefer to spend the dry season (March to November) at the outstations, where they routinely collect and hunt traditional bush foods, and take part in ceremonial activities. During this time, people still regularly drive to Maningrida, to access essential services such as the post office, bank, petrol station and supermarket. At the start of the wet season, before the roads linking the outstations to Maningrida become flooded and unpassable, people typically move back to Maningrida, leaving the outstations empty. Most Indigenous people (including children) living in and around Maningrida are highly multilingual, due to the close contact between the languages of the region and frequent intermarriage. The Kune language, as spoken in Buluhkaduru Outstation, appears to be transmitted very well to young children, as all are able to speak the language fluently, in spite of regular attendance at the local school, where the language of education is English. Over many visits to Buluhkaduru, the author has observed a wide variety of hunting, fishing and bushfood gathering activities, targeting, among others, a handful of fish species (freshwater and estuarine), aquatic turtles, monitor lizards, honey from stingless bees, various yam species and a number of edible fruits.

### Data collection

Prepared stimulus sets were shown to members of an extended family group living at Buluhkaduru Outstation. A total of 13 Kune speakers participated in the study, which represents almost all the adult residents at the outstation at the time of data collection. A larger sample size was not possible because the total number of Kune speakers is itself small, and speakers often travel between widely dispersed settlements. Two stimulus sets were used in this study. The first was a set of plant photographs taken from the book Top End Native Plants [23], which catalogues many common and culturally important plants found at the study site. The second set was audiovisual in nature, and consisted of bird photographs with accompanying recordings of the birds’ calls or songs (if any). Birds were selected on the basis of local occurrence data obtained from the website Atlas of Living Australia [24]. This ensured that the species represented in the stimulus set could indeed be observed in the study area. Bird calls and songs were obtained from the website Xeno-Canto [25], while pictures were sourced from online databases, such as Avibase [26]. This approach has been used successfully in previous ethnobiological studies carried out within the context of a language documentation project (e.g. [27]), and has yielded rich linguistic and cultural information, as well as reproducible results. The plant and bird stimulus sets included approximately 80 and 100 species respectively. It was intended that the stimulus sets be used to investigate naming patterns in multiple language communities of north-central Arnhem Land, and so, species that could be found in both coastal and inland habitats were included in the sets. Therefore, there was no expectation than any single respondent would be able to name all the species in a stimulus set, as at least some of the species would only be found in habitats not normally encountered on his/her traditional lands. Kune speakers, for instance, were not expected to provide names for mangrove plants or coastal birds.

The participants (7 male, 6 female) ranged in age from 19 to 80. Participants were individually shown photographs from each stimulus set, and asked to provide the name of the plant or bird species in Kune, along with any relevant ethnobiological information they might know about that species. The presentation of each bird photograph was accompanied with the playback of an audio recording of the call or song of that species (if available). Participants’ responses were noted in a standard romanized orthography for the Kune language. The proportion of each stimulus set that was accurately named by each participant was calculated (i.e. sorted by participant), as was the number of participants able to name a particular species (i.e. sorted by species). Allowance was made for some degree of dialectal variation between the speech of individual respondents, as the names of some ethnotaxa can vary widely even within the same language group. Participants were also asked briefly about the amount of time they normally spend at the outstation (as opposed to in the town of Maningrida). Non-parametric statistical analyses were carried out through the Social Science Statistics website [28].

## Results

### Variation by age

**Fig. 2:**
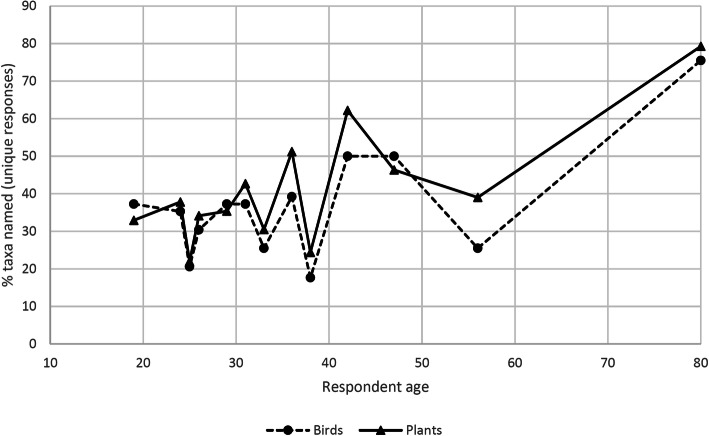
Relationship between age and performance in the plant and bird naming tasks

There was much variation in the total number of plants and birds that could be named by individual participants (Fig. 2). The following analysis takes into account the total number of unique plant and bird names offered by each participant on being presented with the stimulus sets. It does not take into account the accuracy of respondents’ plant or bird identifications. In the case of plant identification, age was significantly, and positively, correlated with the number of unique names recorded from individual respondents (Spearman’s Rho = 0.60, *p* = 0.02). Accordingly, the youngest respondents, aged 19–25 years old, were able to provide unique names for roughly 20–30% of the plants shown to them, whereas the oldest respondent, aged 80, was able to identify close to 80% with unique names. In the case of the bird stimuli, there was no statistically significant correlation between age and the number of names recorded (Spearman’s Rho = 0.34, *p* = 0.24). Here too, however, it was the oldest respondent who was able to name the most birds (around 75%). Consistent with the lack of a significant correlation between age and performance is the finding that some older adults were outperformed by their younger relatives. This is evidenced by the jagged line linking the data points in Fig. 1. A further key finding is that the bird and plant scores correlated very well across respondents (Spearman’s Rho = 0.86, *p* = 0.00012), indicating that individual respondents were able to name plants and birds to a similar degree.

As mentioned above, age did not have a statistically significant effect on the respondents’ ability to name birds. In spite of the significant correlation in the plant naming task, only part of the variation here can be explained by age. This is made apparent by the fact that removing the last data point in the series (that of the 80-year-old speaker; see Fig. 1) renders the correlation for the plant data non-significant (Spearman’s Rho = 0.49, *p* = 0.10). This implies that factors other than respondent age among the younger (19–56 years) respondents must be affecting their ability to name birds and plants from the picture stimuli. Gender is likely to play a role here—it can be hypothesized that men tend to be more knowledgeable about birds than women, and a tendency in this direction can be seen in the current data. However, the low sample size precludes a systematic investigation of gender as a variable.

### Variation by ethnotaxon

**Fig. 3:**
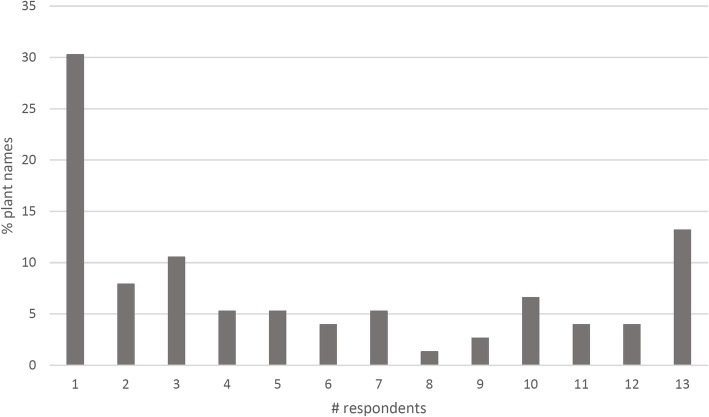
The proportion of plant ethnotaxa named by varying numbers of participants

As predicted, Kune speakers were not able to name plant and bird species associated exclusively with marine or coastal environments. These species are omitted from the following analysis. Looking at the data from the point of view of the number of respondents able to name a certain plant species (Fig. 3), it becomes apparent that only 10 of the plants in the stimulus set (around 13%) were known to all respondents. Unlike the previous analysis (i.e. by age), this analysis takes into account the accuracy of respondents’ plant and bird identifications. The responses of the oldest participant are assumed to be the ‘correct’ identifications, as this person is considered to be the most knowledgeable elder in the community. Indeed, several plant species (23, or 30%) were only identified by this respondent, with the others saying that they did not know the names of these species. Thirty-one plant species were known to at least half (6 or more) of the respondents, representing roughly 41% of the stimulus set. Practically all the plants that were known to all respondents are those that are regarded as a food source by Kune speakers (Table 1), many producing fruit that are easily harvested. Important among these are medium to large trees such as *Buchanania obovata* (green plum) and *Terminalia carpentariae* (wild peach), as well as smaller plants such as *Cassytha filiformis* (dodder) and *Nymphaea violacea* (waterlily).

**Table 1:** Plants ranked according to the number of people able to name them (total *N* = 13)

Scientific name	Kune name(s)^a^	Number of respondents	Recorded uses^b^
*Antidesma ghaesembilla*	djulukkurn, kunj kurlba, djubbi	13	Fruit eaten
*Buchanania obovata*	man-moyi	13	Fruit eaten
*Cassytha filiformis*	burrunburrun	13	Fruit eaten
*Morinda citrifolia*	man-ngukmanj, man-ngukbanj	13	Fruit eaten, medicinal
*Nymphaea violacea*	wayuk, budbarrk	13	Fruit, stem, roots eaten
*Pandanus spiralis*	kun-dayarr	13	Seed, leaf base eaten
*Syzygium eucalyptoides*	bokorn	13	Fruit eaten
*Syzygium potamophilum*	kiddjanjdjanj	13	Fruit eaten
*Syzygium suborbiculare*	djarduk	13	Fruit eaten
*Terminalia carpentariae*	man-mobban	13	Fruit eaten
*Bambusa arnhemica*	man-kole, barakkarl	12	Spear-making
*Ficus virens*	djarnhba	12	Fruit eaten, fibres used to make string
*Melaleuca leucadendra*	kun-kod	12	Bark used for building material, making artefacts
*Acacia difficilis*	man-djoh	11	Seeds eaten
*Cycas angulata*	man-dirnku, ngaddu	11	Seeds eaten
*Livistonia humilis*	djarnkele, djadjak	11	Pith, shoots, fruit eaten
*Callitris intratropica*	man-larru	10	Medicinal bark
*Corypha utan* (formerly *C. elata*)	kurlwirri	10	Growing tip eaten
*Erythrophleum chlorostachys*	man-dubang	10	Medicinal bark, leaves burnt for ‘smoking’ ceremonies, hard wood for construction
*Eucalyptus miniata*	man-balanjdjarr	10	Medicinal bark, seeds eaten
*Flagellaria indica*	bardderdde	10	Fruit eaten, fibres used to make string
*Gronophyllum ramsayi*	kolng	9	Growing tip eaten, leaves used to make artefacts
*Nauclea orientalis*	dubal	9	Fruit eaten
*Terminalia ferdinandiana*	man-marlak, man-manjarr	8	Fruit eaten
*Eucalyptus alba*	kolokkolo, ?man-komborlo, ?warlan	7	?
*Flueggea virosa*	man-korrowon	7	Fruit eaten
*Grewia retusifolia*	man-djotmo, murriddjam	7	Fruit eaten
*Grevillea pteridifolia*	man-bongko	7	Nectar sucked from flowers, leaves used to flavour meat
*Brachychiton paradoxum*	budbud, man-ngarnanj	6	Fruit eaten, fibres used to make string
*Carallia brachiata*	man-wirdu, man-yoku	6	Fruit eaten
*Petalostigma pubescens*	man-bedde	6	Children play with immature fruit*
*Eucalyptus tetradonta*	man-buluddak	5	Bark used for bark paintings, inner bark and leaves are medicinal*
*Cymbidium canaliculatum*	durda, nyarlkkan, djalamardi	5	Sticky sap used as a paint fixative
*Tamarindus indica*	djambang	5	Fruit eaten
*Xanthostemon paradoxus*	man-riyak, ?man-burlu	5	?
*Banksia dentata*	man-limbidj	4	Dried seed pods used to comb hair, can be carried smouldering for long distances to light new fires
*Eucalyptus bleeseri*	man-kalarr, ?man-djuwi	4	Medicinal resin*
*Persoonia falcata*	man-dark	4	Fruit eaten, medicinal inner bark and leaves*
*Sterculia quadrifida*	nawurleb	4	Seeds eaten, medicinal inner bark*, fibres used to make string
*Barringtonia acutangula*	man-manjarr, rdangki	3	Crushed leaves used as fish poison*
*Calytrix exstipulata*	man-barnabbarna, ?man-barndarr	3	Mosquito repellent*
*Capparis umbonata*	man-djiliwirn	3	Fruit eaten, medicinal bark and leaves*
*Cartonema spicatum*	dikkala	3	Edible yam
*Crinum arenarium* (prev. *C. angustifolium*)	kurlumudduk, kolomoddok	3	Poisonous, associated folklore, medicinal bulb and leaves*
*Eucalyptus clavigera*	djanorro	3	Ash from bark mixed with tobacco*
*Ficus racemosa*	warnwarnh	3	Fruit eaten, canoe made from trunk*
*Vitex glabrata*	man-kurndalh	3	Fruit eaten
*Acacia dimidiata*	man-borrelk	2	?
*Casuarina equisetifolia*	djarah	2	?
*Eucalyptus grandifolia*	kolokkolo	2	?
*Eucalyptus polycarpa*	man-bune	2	?
*Pandanus aquaticus*	man-djimdjim	2	Leaf base eaten
*Planchonia careya*	man-wadberr	2	Fruit eaten, medicinal inner bark*, fish poison*
*Acacia auriculiformis*	birlibirlih	1	Bark ash mixed with tobacco*, medicinal leaves*
*Acacia gonocarpa*	man-bulkung	1	?
*Acacia holosericea*	man-merrulk	1	Hard wood for artefacts*, leaves used as soap and fish poison*, edible seeds*
*Acacia platycarpa*	barlarra	1	?
*Alphitonia excelsa*	dird	1	Leaves have saponins*
*Alstonia actinophylla*	namoroddo	1	Canoe made from trunk*
*Amyema sanguineum*	man-djinirrinj	1	Fruit eaten*
*Bombax ceiba*	kordow	1	Making canoes and artefacts*
*Clerodendrum floribundum*	man-molorrk	1	Tap root is edible after roasting (emergency food)*, medicinal uses*
*Eucalyptus ptychocarpa*	man-korlangrlang	1	?
*Eucalyptus ferruginea*	man-dangdang	1	?
*Hakea arborescens*	?man-bardderre	1	Medicinal inner bark*
*Hibiscus menzeliae*	manyalhmanyalh	1	Fibres used to make string,
*Jacksonia dilatata*	?wayarramono, ?wayarramurrngo	1	Medicinal inner bark*
*Leptospermum longifolium*	man-wurrkula	1	?
*Melaleuca argentea*	man-murrmu	1	Bark for building shelters, medicinal leaves*
*Melaleuca cajuputi*	wendelwendel	1	Medicinal leaves*
*Melaleuca dealbata*	djarrkah	1	?
*Melaleuca symphocarpa*	man-domoddomo	1	?
*Mimusops elengi*	dalinga	1	?
*Protasparagus racemosus*	birndiyay	1	Medicinal root
*Tacca leontopetaloides*	karlanj	1	Yam and fruit eaten
*Vigna vexillata*	burlkud	1	Edible yam

The prefixes *man*- and *kun*- are noun class markers in Kune for the vegetal and inanimate classes respectively

Asterisk indicates information from [29], pertaining to unrelated language groups of the Northern Territory; Kune people may not necessarily use these plants in the same way

^a^When more than one name is given, the first is generally the one associated with the people living Buluhkaduru, while the other words may be from neighbouring languages or dialects, which are frequently used by Kune speakers as synonyms

^b^Sources: [29–31], author’s field notes

The plants that were known to only one respondent (invariably the 80-year-old) were more varied in nature, and included species, from large genera, that are similar in appearance (e.g. *Melaleuca* spp., *Acacia* spp.) or plants not regarded nowadays as having any particular use (e.g. *Alphitonia excelsa*, *Hakea arborescens*). Most of the younger respondents did not distinguish between the various species of *Melaleuca* or *Acacia*, whereas they were given distinct names by the 80-year-old respondent (Table 2). Younger speakers tended to label all *Melaleuca* spp. and *Acacia* spp. using the general terms *kun*-*kod* and *man*-*djoh* respectively. For the oldest respondent, the name *man*-*djoh* only applies to the species *Acacia difficilis*. The word *kun*-*kod*, used as a general term by younger speakers for the various *Melaleuca* species, also refers to the papery bark of the trees. A similar, but weaker pattern was seen for *Eucalyptus* spp. While the 80-year-old respondent was able to produce 8 different labels for various species of *Eucalyptus*, the younger people’s responses ranged from 1 to 6 labels.

**Table 2:** Kune names for species of *Acacia*, *Melaleuca*, *Eucalyptus* and *Pandanus*

Scientific name	Kune name	General term
*Acacia auriculiformis*	birlibirlih	man-djoh
*A. difficilis*	man-djoh	
*A. dimidiata*	man-borrelk	
*A. gonocarpa*	man-bulgung	
*A. holosericea*	man-merrulk	
*A. platycarpa*	barlarra	
*Melaleuca argentea*	man-murrmu	kun-kod
*M. cajuputi*	wendenwendel	
*M. dealbata*	djarrkah	
*M. leucadendra*	wendenwendel	
*M. symphocarpa*	man-domoddomo	
*Eucalyptus alba*	kolokkolo	(none)
*E. bleeseri*	man-kalarr	
*E. clavigera*	djanorro	
*E. ferruginea*	man-dangdang	
*E. miniata*	man-baladjarr	
*E. polycarpa*	man-bune	
*E. ptychocarpa*	man-korlangrlang	
*E. tetrodonta*	man-buluddak	
*Pandanus spiralis*	man-dayarr/ kun-dayarr	kun-dayarr
*P. aquaticus*	man-djimdjim	

**Fig. 4:**
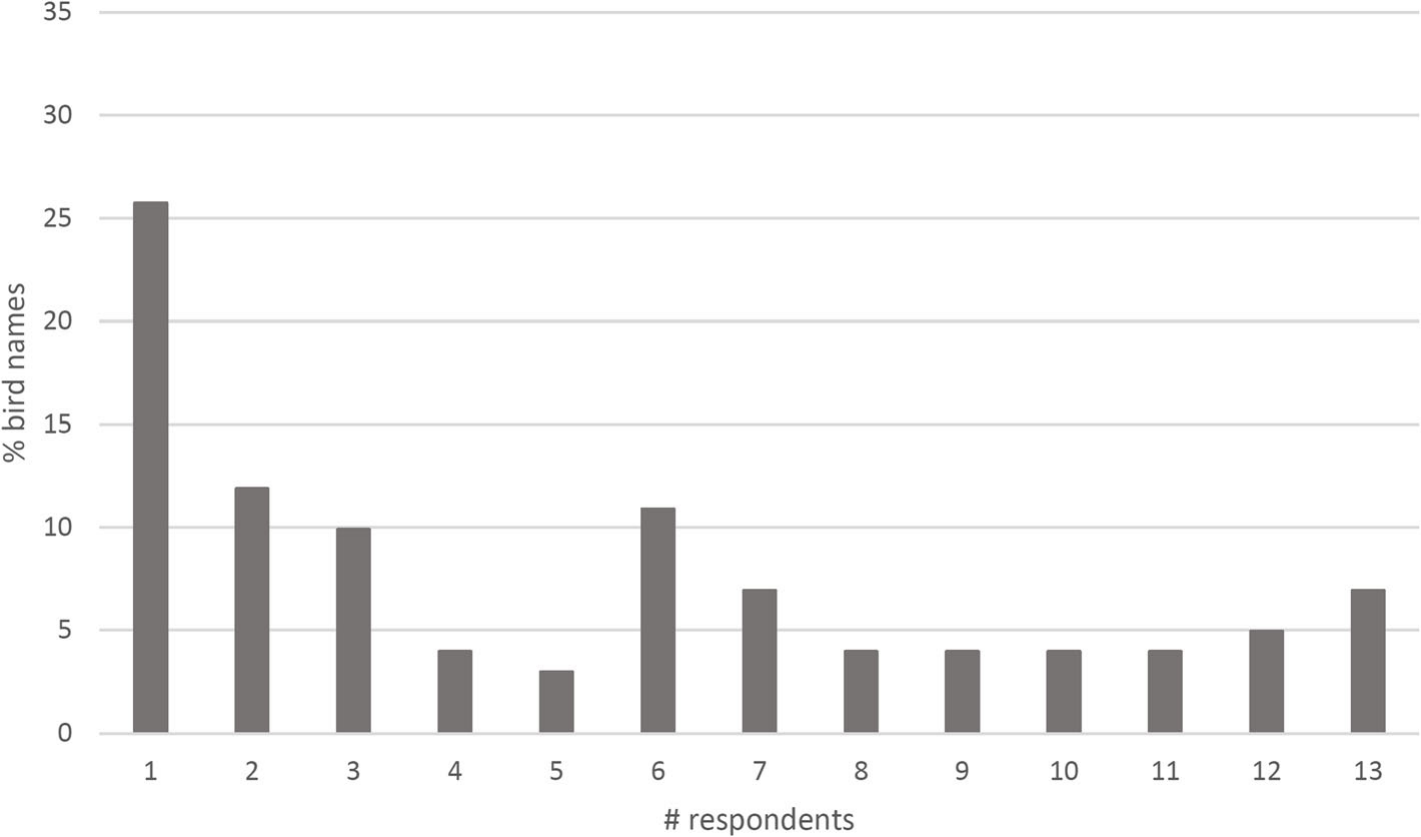
The proportion of bird ethnotaxa named by varying numbers of participants

The quantitative bird data showed a similar pattern overall, with the greatest number (26 of 101 species, or 26%) named by only one respondent, i.e. the oldest (Fig. 4). There was no significant difference between the distributions shown in Figs. 2 and 3 (χ^2^ = 15.3, d.f. = 12, *p* = 0.77). However, the proportion of bird species that were known to all respondents was lower (approximately 7%) than the corresponding figure for plants (13%). Forty-six bird species were known to at least half (6 or more) of the respondents, representing roughly 46% of the stimulus set. The category of birds known to all or nearly all respondents (at least 12 out of the total 13) included large and/or commonly eaten birds, such as the Emu and Magpie Goose, birds with loud, distinctive calls, such as the Torresian Crow and the Blue-winged Kookaburra, or birds with distinctive behaviours, such as the Great Bowerbird (Table 3).

**Table 3:** Birds ranked according to the number of people able to name them (total *N* = 13)

Scientific name	Kune name(s)^a^	Number of respondents	Recorded or potential interactions^b^
Magpie Goose	murnubbarr	13	Eggs and meat eaten (+)
Australian Bustard	benok, walbburrungku	13	Meat eaten (+)
Bush Stone-curlew	kurrubirla, kuwirluk	13	Loud calls heard at night
Torresian Crow	wakwak	13	Totem, songline, loud calls
Emu	wurrbbarn, ngurrurdu	13	Meat eaten (+)
Barking Owl	ngokngok	13	Distinctive call heard at night
Great Bowerbird	djuweh	13	Distinctive nest
Australian White Ibis	karrarla, kalamorn	13	Meat eaten
Blue-winged Kookaburra	korrokkorrow	12	Distinctive evening call
Eastern Koel	djawok, duwoh	12	Distinctive seasonal call
Brolga	ngal-kordow, kodorrko	12	Meat eaten
Australian Pelican	mola, mula	12	Meat eaten (+)
Brown Goshawk	karrkanj, malawirdiwirdi	11	Spreads bushfires, totem
Black-necked Stork	kanjdji	11	Meat eaten, totem
Peaceful Dove	koloddoddok	11	?, common
Rainbow Lorikeet	dedded	11	?, common
Sulphur-crested Cockatoo	ngarradj	10	Meat eaten
Pheasant Coucal	bukbuk	10	Meat eaten
Red Goshawk	karrkanj, marram	10	spreads bushfires, totem
Bar-shouldered Dove	bokodjbokodj	10	Meat eaten
Grey Teal	djilikuyibi	9	Meat eaten
Red-tailed Black-cockatoo	ngarnarrngh, karnamarr	9	Meat eaten (+), eggs eaten
Magpie-lark	marlibrlib, diladila	9	?, common, distinctive call
White-bellied Sea-eagle	mibbarr, makaka	9	?, totem
Australasian Darter	mandangarli, barrakbarrak	8	Distinctive behaviour, meat eaten
Little Corella	ngalelek	8	Meat eaten (+)
Helmeted Friarbird	kawolk	8	Meat eaten
Tawny Frogmouth	kuluyhkuluy	8	Meat eaten
Eastern Great Egret	komorlo	7	Meat eaten
Black-faced Cuckooshrike	widjiwidjik, wirriwirriyak	7	Distinctive call
Zebra Finch	djurrkurl, ninhninh	7	?
Chestnut-breasted Mannikin	djurrkurl, ninhninh	7	?
Rainbow Bee-eater	berrerhberrerh	7	Mythological link
Great Cormorant	bonbon, barrakbarrak	7	Distinctive behaviour, meat eaten
Masked Lapwing	berrebberreb	7	Aggressive, distinctive call
Little Kingfisher	djirrirdirdi	6	Distinctive behaviour
White-bellied Cuckoo-shrike	widjiwidjik, wirriwirriyak	6	Distinctive call
Cicadabird	widjiwidjik, wirriwirriyak	6	Distinctive call
Brown Quail	djirribbidj, djirbbidj	6	Meat eaten (+)
Plumed Whistling-Duck	djirribiyuk, djulukuyibi	6	Meat eaten
Green Pygmy-goose	diwidj	6	Meat eaten (+)
Rufous Owl	ngokngok	6	Distinctive call at night
Royal Spoonbill	murluimurlui, mulunjmulunj, bunberl	6	Meat eaten
Sacred Kingfisher	djirrirdirdi	6	Distinctive behaviour
Southern Boobook	ngokngok	6	Distinctive call at night
Wedge-tailed Eagle	biyangdungkah	6	?, large size
Galah	wirlihwirlih	5	?
Glossy Ibis	birndu	5	?
White-necked Heron	kawarrkkawarrkken, kondo, karladjarr	5	Meat eaten
Black-shouldered Kite	mibbarr	4	?
King Quail	djirrirddih, djirribbidj	4	Meat eaten
Partridge Pigeon	dabbarr, rakul	4	Meat eaten (+)
Radjah Shelduck	karrkarala	4	Meat eaten (+)
White-browed Crake	djinarrarr, djinarradjinarra	3	?
Azure Kingfisher	djirrirddirddih	3	Distinctive behaviour
Pied Imperial Pigeon	rumuh, marlun	3	? possibly eaten
Black Bittern	kondoh, durukmud	3	?
Orange-footed Scrubfowl	ngal-kodjdjorrmi	3	Meat eaten
Nankeen Night-heron	kalkorowk	3	Meat eaten (+)
Red-browed Pardalote	djurdudjurdumun	3	?
Rufous Fantail	djikkiridjdjikkiridj	3	Distinctive behaviour
Grey Fantail	djikkiridjdjikkiridj	3	Distinctive behaviour
Spotted Nightjar	lablab	3	Distinctive call
Pacific Black Duck	ngarnkul, dedjkorrk	2	Meat eaten
Red-winged Parrot	djadberlhberl, weley	2	?
Striated Heron	kulu, durukmud	2	Meat eaten
Large-tailed Nightjar	lablab	2	Distinctive call at night
Rufous-throated Honeyeater	birnhbirndok, djurdudjurdumun	2	?
Blue-faced Honeyeater	yahyih, rolongadji, birdibarlmard	2	?
Gouldian Finch	djurrkurl, ninhninh	2	?
Diamond Dove	korlodohdoh	2	?
Chestnut-quilled Rock-pigeon	borrobborro, dodoro	2	Meat eaten (+)
Northern Rosella	?djikkilirridj, djikkelerinj	2	?
Hooded Parrot	djikkilirridj, djadberlhberl	2	?
Pied Butcherbird	warrhdjird, kobbirdidj	2	Distinctive call
Fork-tailed Swift	yerrelh	1	?
Great-billed Heron	kulu	1	Meat eaten
White-breasted Woodswallow	djerdedjerd	1	?
Pacific Baza	malawirdiwirdi	1	?
Pallid Cuckoo	djirungh	1	Distinctive call
Great Knot	buluwirdwird	1	?
Sharp-tailed Sandpiper	buluwirdwird	1	?
Emerald Dove	dodoro	1	? possibly eaten
Silver Gull	djirrimirla	1	?
Black-tailed Treecreeper	madjirnhmadjirnh	1	Distinctive behaviour
Oriental Cuckoo	djirungdjirung	1	Distinctive call
Black Butcherbird	warrhdjird	1	Distinctive call
White-faced Heron	kawarrkawarrken	1	Meat eaten
Pied Heron	mungkulmungkul	1	Meat eaten
Dollarbird	rdewrdew	1	Distinctive call
Brahminy Kite	djurddjurd	1	Totem
Comb-crested Jacana	kodabbirl, djinarrarrdjinarrarr	1	Distinctive behaviour
Square-tailed Kite	malawirdiwirdi	1	?
Crested Pigeon	wirrirwirrir	1	? common
Green Oriole	kodkangardidjbun	1	Distinctive call
Mangrove Golden Whistler	nyuridj	1	?
Varied Lorikeet	djurrih	1	?
Banded Fruit-dove	lumbuk	1	Meat eaten
Red-backed Button-quail	merhmerh	1	? possibly eaten
Masked Owl	yerinj	1	?
Eastern Grass Owl	yerinj	1	?

The prefix *ngal*- is a noun class marker in Kune for the feminine class

(+) indicates information from [31], and refers to birds that are considered ‘staple’ or ‘important’ food sources for at least part of the year; this information was obtained from people in west Arnhem Land, who speak the related languages Gundjeihmi and Kinwinjku, but Kune people may not necessarily consume these birds to the same extent

^a^When more than one name is given, the first is generally the one associated with the people living Buluhkaduru, while the other words may be from neighbouring languages or dialects, which are frequently used by Kune speakers as synonyms

^b^Sources: [30, 31], author’s field notes

## Discussion

The people who took part in this study are all native speakers of Kune. Most of the respondents routinely spend the dry winter months of each year ‘in the bush’, i.e. at outstations such as Buluhkaduru, and have an intimate knowledge of the landscape and place names associated with this location. This includes the names and locations of important water bodies, ritual grounds and sacred sites, some of which are to be avoided because of cultural taboos. Members of a family group frequently discuss the location and availability of traditional foods in around the outstation, and organize regular trips to nearby lakes and creeks for hunting and foraging trips, on which fish, turtles and other aquatic reptiles are caught and eaten. Whenever the author accompanied a foraging group on such trips, Kune people would always point out the various foods, along the walking track or at the destination, that were in season; often young children would point out a clump of *mayaddja* grass (*Heteropogon triticeus*), and instruct the author to chew on the stem to obtain the sweet juice inside. At other times, they would make a detour to a location known to be home to a stand of *man*-*mobban* trees (*Terminalia carpentariae*); once again, the children would explain to the author that the late dry season (around September) was the time to pick the sweet fruit from these trees. Younger men often go on hunting trips (nowadays with a rifle), and are expert trackers of wallabies and buffaloes, whereas men and women of all ages take an active and enthusiastic interest in looking for stingless beehives.

These observations appear to be at odds with the findings of the current study, primarily with the result that over half the plants and birds in the stimulus sets could not be named by most respondents. The younger people who took part in this study can undoubtedly name, identify and use many more ethnotaxa than the ones included in the stimulus sets, but it is nevertheless surprising that so many supposedly common plants were not known to many participants. One compelling explanation is that the type of TEK investigated in the present study was of a very specific nature, characterizable as theoretical ‘knowledge’, as opposed to practical ‘skills’ (cf. [19]). In particular, linguistic knowledge (i.e. the names of plants and animals) was chosen as the object of inquiry, because of its relation to the central question of the study—does transmission of language equate to transmission of TEK? Based on the data presented here, the answer appears to be that it does not. However, an important caveat is that TEK can be measured in a number of ways, and no single index can provide a complete picture of an individual’s knowledge, as it invariably spans several domains [32].

Despite the small number of respondents, some interesting patterns are evident in the plant and bird naming results. Three of these patterns are discussed in this and the following sections: the relationship between naming performance and respondent age, the difference between respondents’ ability to name plants and birds and differences in the salience of various plant and bird ethnotaxa, as reflected in participants’ ability to recognize and name them. Positive correlations between age and ethnobiological knowledge have been noted in many rural, minority communities around the world (e.g. [33–35]), but such a pattern was not seen to a convincing degree in the present dataset. Nor was it the case that knowledge levels plateaued in young adulthood (in a person’s 30s), as suggested by Koster et al. [36], because none of the younger participants (aged 19–56) came close to the performance of the 80-year-old participant, and some older Kune speakers were even outperformed by their much younger relatives (Fig. 2). Further discussion on this issue can be found in the section ‘Personal life history and TEK’. Comparable studies carried out with Australian indigenous communities are rare, but a TEK documentation project of the Kija and Jaru languages of the Kimberleys (also in northern Australia) showed a strong correlation between TEK levels and language proficiency [37]. Both Kija and Jaru are highly endangered, and for Kija at least, the youngest fluent speakers were in their forties or fifties at the time of the study. Accordingly, the most proficient speakers tended to have the most TEK. The results from the Blythe and Wightman study are not directly comparable to the current Kune study, because of the very different language endangerment situation. Older Kune speakers would probably agree that their younger relatives possessed less TEK than members of their own generation, akin to the older respondents in [10, 11]. However, this cannot be blamed on an insufficient grasp of Kune, as the language is being transmitted to young children.

An interesting difference between the plant and bird naming results was the positive correlation with age for the plant data only (as long as the oldest respondent’s data point was included). A related observation that sets the two apart is the higher score for plant names, compared to birds, for the majority of respondents. Figure 2 shows that a majority (9 of 13) of respondents were able to name a greater proportion of plants than birds, often by a large margin. One respondent (the 25-year-old) had nearly identical scores for both, and only three respondents scored better at naming birds. It is possible that the differences in the level and intensity of interactions with birds and plants are responsible for the overall better performance in the plant naming task. Perhaps the same explanation could account for the overall lower performance in bird-naming, as well as the reduced ability of respondents to name plants and birds that are no longer eaten or otherwise used (Tables 2 and 3). A utilitarian or adaptationist explanation (cf. [38]) could account for both observations; although Hays first proposed this approach to account for patterns in ethnobiological classification, its central idea could be modified to state that people adapt to their current circumstances by preferentially talking about (and naming) those organisms that are still relevant to those circumstances. It is the names of these organisms that are acquired by children in the community. A more in-depth discussion of Kune folk classification is beyond the scope of this paper, but some indication of the lifestyle changes that have occurred in Kune society, which might explain the reduced transmission of some ethnotaxa names, is provided in the following section.

### Lifestyle change and TEK

Proximity to the town of Maningrida allows many indigenous groups the opportunity to access cash and services, such as banking, government aid, a clinic, a school, an airstrip and a post office. Two supermarkets and a small general store provide access to a wide range of mainstream, urban foods, shipped in by barge from the Northern Territory’s capital, Darwin. The author has accompanied the Buluhkaduru family on numerous shopping trips, and noted that the most commonly purchased staples were flour, butter, sugar, milk powder and tea. The flour is used to make damper—a kind of bread prepared over a campfire—which is eaten with butter, while tea with milk and sugar is the staple hot drink consumed every morning. Tinned corned beef is a much loved food item, but one that is not consumed very frequently, due to its high price. Frozen beef and chicken are regularly purchased from the supermarkets, to supplement the fish and bushmeat available at the outstation. Another important source of meat is the local ranger group called Bawinanga Rangers (formerly Djelk Community Rangers). Among their many land management activities is the control of the feral buffalo population through culling. Animals that are shot are butchered, and the meat is shared with nearby outstations. For this reason, the traditional owner of Buluhkaduru normally has a stock of buffalo meat in the freezer he keeps in his house.

Altman [39] describes three phases in the history of north-central Arnhem Land, while focusing on the experiences of the Kuninjku people, who speak a language closely related to Kune. These were ‘precolonial’, when indigenous people of the region were nomadic, and entirely dependent on hunting and gathering prior to the establishment of a government township at Maningrida in 1957, ‘colonial’, between 1957 and 1972, when indigenous people were settled in Maningrida, and expected to assimilate into mainstream Australian culture, and ‘postcolonial’, marked by a change in government policy towards self-determination, and a general movement of indigenous people towards outstations and traditional lifestyles. For the Kune at Buluhkaduru, food security is higher nowadays than in the recent past, when people had to be more self-sufficient, and the bulk of the family group’s nutrition was derived from hunting and gathering. In spite of the abundance of food available at Buluhkaduru at certain times of the year, such outstation locations are invariably associated with ‘a degree of seasonal precarity’, as ‘making a living out in the bush during the wet seasons is difficult because of seasonal flooding and inaccessibility of wildlife’ ([40], p. 172). The older people who currently reside at Buluhkaduru speak of frequently going hungry when they were young, a situation that started to change when the Bawinanga Aboriginal Corporation in Maningrida initiated a system of regular food deliveries by truck to pre-determined locations in the late 1970s.

It is likely that prior to achieving regular access to mainstream foods, Kune people had to rely on a much larger array of plant and animal species for food, medicine and raw materials than they do today. Table 1 shows that the vast majority of plant species included in the stimulus set have potential uses for indigenous people. Note, however, that most of the plants with edible fruit (i.e. a resource that is relatively easy to find and gather) could be identified and named by the majority of participants. The plants further down the table, that could be identified by only one, or a handful of, participants, tended to be used more for medicinal, construction or other purposes. It is likely that potential famine foods, that often require arduous or time-consuming labour for a small reward, are among the first plants to be overlooked by younger people. An example of this would be *Acacia holosericea*, the seeds of which can be ground to make flour, which is in turn baked to produce damper [29]. While this species can be found at Buluhkaduru, and was called *man*-*merrulk* by the oldest respondent, the author has never seen any Kune person collect or consume the seeds of any *Acacia* species. Smith also notes that certain species of *Acacia*, such as *A*. *holosericea* and *A*. *auriculiformis*, provide treatments for ailments such as skin sores, and contain saponins which help in washing hands or clothes; the leaves of these plants can also be crushed and thrown into small water bodies to stun fish. The therapeutic uses of such plants have nowadays been taken over by modern medicines and artificial soaps, whereas fishing is done almost solely with a hook and line. Moreover, specialist knowledge about the medicinal or magical uses of this and other plants may have resided within ‘clever men’, an Aboriginal English term for what anthropologists call ‘sorcerers’ or ‘medicine men’ [41]. Most published accounts on ‘clever men’ from the Northern Territory focus on people who have passed away (see [42] for some examples), and the likelihood is high that few, if any, such people are currently practicing their art. It is perhaps not surprising then, that younger Kune people no longer distinguish between the different species (and ethnotaxa) of *Acacia*, preferring instead to use the umbrella term *man*-*djoh* (which for older people, is the name for *A*. *difficilis* only). Incidentally, *man*-*djoh* was probably chosen as the prototypical ethnotaxon because it is the most common local *Acacia*, such that the place name Buluhkaduru (which is actually *buluh ka*-*duru* in the neighbouring Rembarrnga language) can be translated as ‘*A*. *difficilis* stands (here)’.

Another example of the use of an ethnotaxon label being extended to include a distinct ethnospecies is that of *Pandanus spiralis*, the seed kernels of which can be eaten raw or roasted. The tough outer covering of the fruit has to be removed first, and the seeds seem to be eaten only infrequently nowadays. A related species is *Pandanus aquaticus*, the seeds of which are not consumed. Two of the oldest males who took part in the current study provided different names for *P*. *spiralis* and *P*. *aquaticus*—these were *man*-*dayarr*/*kun*-*dayarr* and *man*-*djimdjim* respectively. All other respondents, however, named both species with the name label *man*-*dayarr*/*kun*-*dayarr*, although they knew that only the seeds of *P*. *spiralis* were edible. Here too, it seems that a traditional distinction between two ethnotaxa is slowly being lost due to lack of use.

There are significant exceptions to the trend of disuse of a plant leading to the loss of the relevant name from the active lexicon of individual speakers. One such example is the cycad ‘palm’ *Cycas angulata* (and possibly also *C*. *armstrongii*), which was accurately named by 11 of 13 respondents, even though it is rarely eaten nowadays. The toxic seeds need to be prepared appropriately over several days to render them edible, and the end product, again a kind of damper, has an unpleasant smell. However, the name remains in current use, probably because of the importance of the damper in ceremonial exchanges [43], where large numbers of people need to be fed [44]. The observation by earlier researchers [31] that the consumption of cycad seeds was never really widespread in western Arnhem Land (where Buluhkaduru is situated) makes it all the more remarkable that the name of this plant is still widely known among younger people. One well-known plant that is not even found on Kune traditional lands is the cabbage palm *Corypha utan*, which was identified and named by 10 participants (Table 1). This tree only grows in eastern Arnhem Land [24], within the territory of the unrelated Yolngu languages, but is known to Kune speakers because of its totemic significance.

It is a more difficult matter to offer an explanation for the loss of certain bird names from the active lexicons of some respondents. As with the plants, many of the well-known birds in this study are frequently consumed, or are considered to be desirable food items. In Table 3, most of the birds (8 out of 12) considered to be ‘staple’ or important’ foods (*sensu* [31]) could be named by six or more participants, while only 4 appeared to be largely unknown. Foremost among the well-known edible birds is the Magpie Goose, whose flesh is sought after whenever flocks gather on swamps and other bodies in the wet season; others include the Emu, Australian Bustard, various ibises and storks, Brolga and perhaps also the Australian Pelican. It is likely that many other birds—such as the Pheasant Coucal, and perhaps smaller species including pigeons, cockatoos and parrots—were hunted for their flesh, or had their nests raided for their eggs. Many other birds could also have been hunted and eaten in times of need, but it is difficult to say anything conclusive on this subject, as not much has been published on the interactions of Australian indigenous people and the smaller avifauna in their environment, and it is unlikely that any of the respondents in this study would have regularly consumed smaller birds in the past. Boys and young men at Buluhkaduru often hunt smaller birds for sport with a slingshot, but rarely do people nowadays make a concerted effort to travel to a location where, for instance, large waterbirds (other than Magpie Geese) might be found.

Some birds may be known to many respondents due to their distinctive calls and/or behaviours or their ritual/totemic associations. The Bush Stone Curlew, Barking Owl, Eastern Koel and Blue-winged Kookaburra are prominent within the former category, as they are well known to call loudly at certain times of the day or of the year. The Great Bowerbird makes unusual, decorated nests on the ground, while the Brown Goshawk (and possibly also other species, such as the Red Goshawk) is known among all Arnhem Land communities for picking up burning twigs and setting bushfires in new locations, so as to flush out its prey consisting of reptiles and small mammals [45]. These birds are also celebrated in myth and ceremony, as is the Torresian Crow, which is an important totemic animal for the neighbouring Marrangu Djinang people [46]. Ultimately, however, it is likely that many bird names are being forgotten, or not being learnt, by younger people because they are no longer relevant to people’s lives in modern times. The observation that a smaller proportion of bird species, compared to plant species, were known to all participants suggests that people are interacting with plants to a greater degree than with birds.

As with the plants, there was some tendency among younger participants to generalize the name for a single well-known ethnotaxon to include other, similar-looking birds. This happened most frequently with the name *dedded*, which the oldest participant used to label the Rainbow Lorikeet only. Among the responses of the younger participants, *dedded* was offered as a name for a variety of colourful birds, such as parrots, lorikeets and rosellas. The labels *korloddoddok* (Peaceful Dove) and *bokkodjbokkodj* (Bar-shouldered Dove) were also frequently used by younger participants to label other pigeons and doves.

Although mammals were not included in the current stimulus sets, it is very likely that younger Kune speakers—especially young adults and children—would struggle to name all but the largest mammals that are present on their lands. While assisting an elderly Kune speaker in a language and culture class at Maningrida College in 2014, the author noted that the children in the mixed-age classroom (the eldest child being around 14 years old) labelled all local kangaroo species with the general term *kunj* ‘wallaby/kangaroo’, instead of using the highly specialised terminology that would presumably be known to their parents. Kune and related languages recognize several kangaroo and wallaby ethnotaxa, and have distinct words to describe the different life stages and gender of each kangaroo/wallaby species, as well as distinct verbs to describe their hopping action [47]. However, it appeared that the children in that classroom were hearing many of these words for the first time from their elderly teacher. A reduction in the consumption of wild meat in modern times is probably responsible for this phenomenon, but equally responsible is the local extinction of a number of small mammals from around Buluhkaduru, and Arnhem Land in general. External factors such as more intense bushfires, prior to Indigenous-style land management that started in 2006 [48], and the arrival of invasive species such as the cane toad (*Bufo marinus*) have led to the disappearance of once common species like the Northern Quoll (*Dasyurus hallucatus*) and the Northern Hopping Mouse (*Notomys aquilo*). Both species are officially listed as endangered, with the IUCN Red List of Threatened Species even stating that the latter may no longer occur on the Australian mainland [49]. No wonder, then, that most Kune children will have never seen, and may probably never see, the animals that their elders once called *yulukyuluk* and *kidjikidjidayhdayh.*

The above discussion suggests that knowledge of different groups of ethnotaxa may be affected by different factors. The names of certain groups of plants may not be learnt by younger speakers because their uses are no longer relevant to modern living. Certain bird names might not be passed on because they were never important to the community, either in utilitarian terms, or from a ritual/ceremonial point of view. If people of a community spend less time nowadays on traditional lands, or take part in fewer hunting or foraging activities, that would present fewer opportunities to see certain birds, and talk about them. Finally, certain small mammals may simply have gone locally extinct due to environmental degradation, making them no longer salient to young language learners, regardless of how much time they spend carrying out traditional activities. Future studies should therefore aim to investigate people’s TEK on a range of taxa, to uncover community- and location-specific differences in the transmission of TEK.

### Personal life history and TEK

The small sample size investigated in this study provides a good opportunity to consider the experiences and traits of individual participants, as opposed to making generalisations about entire communities. During the naming tasks, practically all participants said at one time or another that they had once known the name of a plant or bird, but could no longer remember it. Often, they were able to provide supporting information—a physical description of the organism, its habitat, traditional uses or an associated cultural belief or folktale—that proved that they were indeed thinking about the right plant or animal, and had merely forgotten its name. An example from among the plants is *Grewia retusifolia*, a small shrub that produces small, edible fruit, and is locally known as *murriddjam* or *djodmo*. The name of this plant was known to seven participants, but a further four said that while they had eaten this fruit in their childhood, they could no longer remember the name. An even more striking example is that of *Crinum arenarium*, which only three people could name. A further seven participants had seen the plant before, and even knew the local belief that touching it could cause a person to be struck by lightning, but could not remember the Kune name *kurlumudduk*. The same phenomenon was seen to a lesser degree in the bird naming task. For instance, some people could accurately describe the behaviours of birds such as the Masked Lapwing and the Comb-crested Jacana, but could not name them.

While it is understandable that people occasionally forget information learnt in childhood, it was a surprise to see some adults in their thirties and even fifties being able to name far fewer ethnotaxa than much younger participants. Anecdotal evidence obtained during discussions with individual Kune speakers revealed a wide range of life histories with the potential to impact the acquisition of TEK. Figure 2 shows that two participants (aged 38 and 56) were able to name only 18% and 25% respectively of the birds shown to them, whereas a 19-year-old relative achieved a score of 33% for the same task. Similarly, a 42-year-old was also able to outperform the 56-year-old by a large degree, in both the plant and bird naming tasks. The cases of the 38-, 42- and 56-year-olds will be briefly considered below, as their divergent life histories seem to be having an impact on their performance in this study. The gender of these participants is left ambiguous in the discussion below to protect their identity.

Both the 38- and the 42-year-old individuals would have been born during the ‘postcolonial’ times (*sensu* [39]), and would have had the opportunity to spend their childhood on traditional lands, while relying to a large extent on traditional means of subsistence, supplemented by occasional food deliveries from town. However, the 38-year-old has been working in an office job in Maningrida for the past few years, and is therefore required to live in town for most of the year. Although s/he grew up in the bush, s/he nowadays visits Buluhkaduru outstation for only a few weeks every dry season. This person frequently stated during the naming tasks that s/he had either eaten a particular plant food as a child, or had seen or heard a particular bird, but could no longer remember the names for these ethnotaxa. The 42-year-old, on the other hand, grew up in a Rembarrnga- and Kune-speaking community to the east of Buluhkaduru, in the company of siblings who are highly regarded nowadays for their bush knowledge. S/he has been married to a respected community elder, who is also a highly skilled bush person, and it is likely that s/he performed well in the naming tasks thanks partly to these influences. S/he also lives at Buluhkaduru for extended periods of time every year with the family group. Unlike the earlier two participants, the 56-year-old began living at a Christian mission from a young age. S/he would have been born during the ‘colonial’ period of Arnhem Land (*sensu* [39]), and would have faced strong pressure to assimilate to a mainstream Australian way of life. This person speaks very good English (alongside fluent Kune), but feels that his/her grasp of TEK is not as good as that of his/her relatives, due to the time spent away from the bush. Finally, it is worth mentioning that the 80-year-old (note that this is an approximate age, as the person’s exact date of birth is unknown) who performed the best in the naming tasks would have grown up in ‘pre-colonial’ Arnhem Land, when contact with mainstream Australian culture was minimal. This person recalls hiding in the rock country to the south of Maningrida during the final days of World War 2, and living off the land in the company of knowledgeable indigenous elders.

Such anecdotes paint a compelling picture of the possible ways in which a person’s life history might affect the current state of their TEK. In general, it seems that time spent away from traditional lands, close family ties with knowledgeable individuals and reduced involvement in traditional activities (due to an intensive western education, for example) can increase or decrease a person’s knowledge of particular domains of TEK. Ideally, a longitudinal study with a larger sample size would be required to explore this phenomenon adequately. A similar phenomenon was reported by [37] in their Kija and Jaru TEK documentation project in northern Australia. The authors concluded that a person’s upbringing has an important effect on his/her level of TEK, with more knowledgeable people having grown up in a Kija-only community, where traditional ceremonies were conducted annually, instead of the alternative cattle station, which was home to many language groups (which promoted the use of Kriol for communication), as well as a Presbyterian mission and school.

## Conclusions

This study has investigated a tiny part of the TEK of Kune speakers, and found that there are signs of a weakening of knowledge transmission in two domains. It is clear from previous studies (such as [31]) and the author’s own interactions with Kune speakers, that many plant species potentially known to, and used by, Kune speakers were not included in the stimulus sets. The most significant group omitted from this study is that of the various yams produced by a range of local species; while these plants may no longer be staple foods, they are nevertheless eagerly sought after, and dug up whenever an opportunity presents itself. Nevertheless, it is noteworthy that younger Kune speakers, who are otherwise fluent in their mother tongue, are no longer able to recall the names for a number of common plant and bird ethnotaxa.

The findings also suggest that there are notable differences in the extent to which two domains of TEK are transmitted to younger speakers. Overall, there seems to be a relationship between lifestyle change (in particular, changes in diet) and the transmission of knowledge, as more younger people were able to preferentially name those plants that were still consumed regularly. The transmission of bird names was found to be less robust than that of plant names, as a far smaller proportion of bird species (compared to the plants) was known to all respondents. This could be due to the declining relevance of certain bird ethnotaxa in the everyday lives of Kune speakers. Differences in the life histories of individual respondents may also be able to explain some of the patterns seen in the data, but this possibility needs to be investigated further, with other language groups. As suggested by Gomez-Baggethun and Reyes-Garcia [50], TEK change can involve the gain of new knowledge as well as the loss of pre-existing information; in future research, it would be interesting to investigate the ways in which Kune people are adapting their TEK to cope with their changing lifestyles and outside influences. Further studies will investigate neighbouring indigenous languages, as well as minority languages in other countries, to determine whether there are any systematic cross-linguistic patterns linking language endangerment and TEK endangerment. Naturally, these studies should involve a larger sample of respondents; this would allow not only a targeted comparison between males and females (male respondents might be expected to perform better with bird naming overall, for instance) but also the inclusion of other types of organisms, such as reptiles, mammals, fish and invertebrates. Comparisons with language communities affected by varying levels of endangerment should result in practical outcomes relating to documentation, maintenance and revitalization efforts. If language and TEK endangerment are positively correlated, then an intervention to halt language loss should also have the effect of safeguarding TEK. However, in the absence of a positive correlation (because TEK and language maintenance are being affected by different factors in a particular community), interventions that only focus on language (such as bilingual education programs in schools or language documentation projects) may not actually help stop the decline of TEK in a community facing language loss. Conversely, a community exhibiting high linguistic transmission may in fact be suffering from TEK loss; such a problem might not be detected by linguists until it is too late.

A further outcome of this study is the identification of those ethnotaxa that are more vulnerable to the effects of TEK and language loss. The responses shown in Tables 2 and 3 illustrate the unequal nature of TEK awareness for different ethnospecies. In particular, over half of the plants and birds in the stimulus sets were known to 5 or fewer respondents (out of a total of 13). The Kune speakers who took part in this study expressed their concerns regarding TEK loss, and appeared eager to take steps to remedy the situation. One young man thanked the author for ‘showing him how much he had forgotten’, and even asked if he could keep the bird stimulus pictures for further study. On the author’s last day at Buluhkaduru, an impromptu TEK learning session took place: some children were going through the album of bird pictures, and they were soon joined by a couple of adults, including the 80-year-old, who proceeded to teach the children the names of the birds. Future efforts to safeguard and/or revitalise language and TEK among Kune speakers could focus preferentially on the vulnerable ethnotaxa identified in this study, by making them feature more prominently in educational resources or in ‘learning on country’ sessions during school hours. Indigenous elders could also be made aware of such imbalances in the transmission of TEK, to enable them to focus on teaching the names and associated TEK of those ethnotaxa that are not being acquired, or forgotten, by younger people.

## Data Availability

All data generated or analysed during this study are included in this published article.

## Abbreviation

TEK: Traditional ecological knowledge
